# Increased levels of vascular endothelial growth factor in the aqueous humor of patients with diabetic retinopathy

**DOI:** 10.4103/0301-4738.67042

**Published:** 2010

**Authors:** Kocabora M Selim, Durmaz Sahan, Taskapili Muhittin, Cekic Osman, Ozsutcu Mustafa

**Affiliations:** Vakif Gureba Education and Research Hospital, Ophthalmology Department, Istanbul, Turkey

**Keywords:** Diabetic retinopathy, macular edema, retinal ischemia, vascular endothelial growth factor

## Abstract

**Purpose:**

This study aims to investigate the levels of aqueous vascular endothelial growth factor (VEGF) in diabetic patient groups in comparison to normal subjects, and to correlate elevated VEGF with the severity of diabetic retinopathy (DR).

**Materials and Methods:**

Aqueous samples were obtained from 78 eyes of 74 patients undergoing intraocular surgery and they were examined by the enzyme-linked immunosorbent assay. Color photographs, optical coherence tomography scans, and fluorescein angiography were used to evaluate patients preoperatively.

**Results:**

A strong statistical correlation was found to exist between the level of aqueous VEGF and the severity of DR (*P* < 0.001), whereas, the VEGF levels in a control group and a diabetic group without DR were not significantly different (*P* = 0.985). Aqueous VEGF levels were significantly elevated in patients with proliferative DR (PDR) as compared to the control group (*P* < 0.001), to diabetic patients without retinopathy (NDR) (*P* < 0.001), and to diabetic patients with nonproliferative DR (NPDR) (*P* < 0.001). The aqueous VEGF levels were significantly higher in patients with active PDR than in those with quiescent PDR (*P* = 0.001). On the other hand, a statistically insignificant (*P* = 0.065) correlation was found between elevated aqueous VEGF and the presence of macular edema in the NPDR group.

**Conclusions:**

VEGF was elevated in the aqueous humor of patients with DR compared to that in normal eyes. The aqueous VEGF level had a strong correlation with the severity of retinopathy along with a statistically insignificant difference in macular edema.

Diabetic retinopathy (DR) is the most common complication of diabetes mellitus and one of the main causes of blindness in the world, especially in developed countries.[1,2] Proliferative diabetic retinopathy (PDR) and diabetic macular edema (DME) are the most common aspects of DR leading to visual deterioration.[1,2] Various cytokines, including the vascular endothelial growth factor (VEGF), have been identified as playing roles in the pathogenesis of DR.[1–4] VEGF that was first discovered as a vascular permeability factor is specifically a mitogenic cytokine for vascular endothelial cells. Retinal hypoxia / ischemia is the basic stimulus leading to the marked ‘upregulation’ and ‘increase’ of VEGF locally, and therefore, plays a major role in the progression of DR. Increased VEGF interacts with its two tyrosine kinase receptors on the retinal vasculature resulting in new vessel formation and also in the disruption of the internal blood retinal barrier. The main causes of moderate or severe visual loss in DR are macular edema and intraocular hemorrhage as consequences of the VEGF-mediated pathology.[1–4]

The present study was carried out to investigate the relationship between VEGF levels in aqueous humor and the severity of DR, and also to compare the results of VEGF levels in subjects without diabetes.

## Materials and Methods

Undiluted aqueous fluid samples were obtained from 78 eyes of 74 patients undergoing intraocular surgery. Forty-two subjects were female (44 eyes) and 32 male (34 eyes) with a mean age of 65.2 ± 10.4 years. Sixty-nine eyes had undergone cataract surgery with phacoemulsification and nine eyes had undergone pars plana vitrectomy. Blood samples from all diabetic patients were taken 24 hours prior to surgery for HbA1c, glucose, and C-reactive protein (CRP) measurements.

Fifty-six of the patients had diabetes mellitus and 18 patients were healthy subjects without diabetes or any other systemic disease. All the patients were carefully examined by slit-lamp before surgery and detailed fundus examinations were made by using a contact fundus lens. Color fundus photographs, fluorescein angiograms (FFA), and macular scans with time-domain optical coherence tomography (OCT) were taken routinely one week before surgery.

The exclusion criteria included: (1) Previous intraocular surgery or laser photocoagulation within the past six months; (2) Associated uveal or retinal pathology other than DR; (3) Glaucomatous eyes; (4) Eyes with ‘rubeosis iridis’; (5) Vitrectomized eyes; (6) Previous intravitreal injection of steroid or an anti-VEGF agent.

The study subjects were divided into four groups:

Group A was the control group consisting of 18 eyes of 18 non-diabetic patients.

Group B consisted of 14 eyes without DR (NDR) of 13 diabetic patients.

Group C consisted of 27 eyes with nonproliferative diabetic retinopathy (NPDR) of 25 diabetic patients. This group was divided into two subgroups: C1 (14 eyes) without clinically significant diabetic macular edema (DME) (CS-DME) and C2 (13 eyes) with CS-DME. Eyes with significant macular thickening had one of the focal, diffuse or mixed type of DME. No vitreomacular traction on OCT and macular ischemia on FFA were detected in these 27 eyes.

Group D consisted of 19 eyes with PDR of 18 diabetic patients. This group was also divided into active (Subgroup D1 = nine eyes) and quiescent (Subgroup D2 = 10 eyes) PDR subgroups. PDR was considered to be active if the neovascular vessels were perfused and quiescent in the presence of non-perfused or / and gliotic vessels or if the previous active proliferation had significantly regressed.

Written informed consent was obtained from each patient at least 24 hours prior to surgery. The study was performed following a protocol that was approved by our hospital’s institutional ethics committee, conforming to the ethical principles of the Helsinki Declaration.

Collection of aqueous samples was conducted in the operating theater under sterile conditions and just prior to intraocular surgery. Samples (0.1 – 0.2 ml) of aqueous humor were collected in sterile tubes by way of limbal anterior chamber puncture with a 27 gauge needle of a 1 ml insulin injector. These samples were then stored frozen at –80°C within a few minutes of collection.

The concentration of the VEGF 165 isoform was measured by enzyme-linked immunosorbent assay (ELISA) by using a kit for human VEGF (Invitrogen U.S.A.-BioSource^®^ Human VEGF Immunoassay kit Catalog # KHG0112 / KHG0111).The linear range of detection was 5 – 1500 pg / ml for the assay.

The Kruskal-Wallis test was used to evaluate the variation and difference between groups for each variable, and the Mann-Whitney test was used to compare the groups for one variable. Pearson correlation analysis was used to measure the strength of the relationship between the variables. The 95% confidence intervals of the tests were determined; *P* < 0.05 was accepted as statistically significant.

## Results

**Figure 1:**
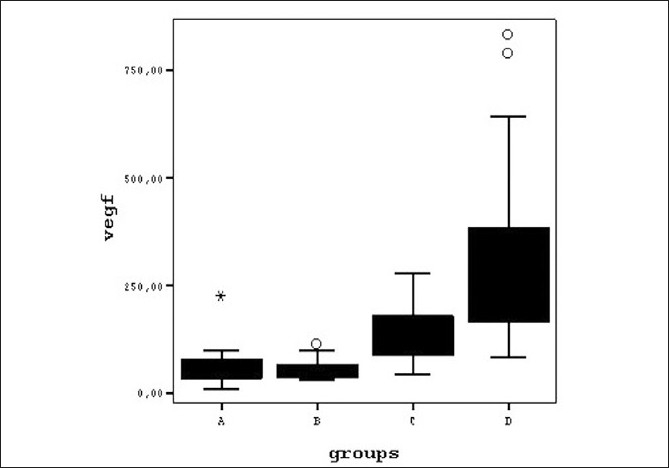
Diagram graphically displaying the differences between the aqueous VEGF levels of study groups on boxplots A = Control Group, B= NDR Group, C= NPDR Group, D= PDR Group

The mean ages of the four subject groups and the biochemical laboratory results are summarized in Table 1. Variance analysis of the groups was done by using the Kruskal-Wallis test. The four groups showed no significant differences with respect to age, blood glucose, HbA1c, and CRP levels. However, the Kruskal-Wallis test indicated a significant difference between the VEGF levels of the four groups (χ^2^ K-W = 42.415; SD = 3; *P* < 0.001). The Mann-Whitney U test using Bonferroni adjustment (statistical significance defined as *P* < 0.05/4) showed significant differences between all groups except between Group A and Group B [Table 2 and Fig. 1]. The aqueous level of VEGF was significantly elevated in the samples from Group D (patients with PDR) (318.6 ± 238.6 pg/ml [83.0 - 836.1] as well as in samples from Group C (patients with NPDR) (133.3 ± 58.5 pg/ml [43.4 – 279]) when compared with those from Group B patients (diabetic without retinopathy) (56.5 ± 27.5 pg/ml [28.3 – 114.2]) and Group A patients (non-diabetic control patients) (62.6 ± 48.8 pg/ml [8.4-228]); whereas, there was no significant difference between the aqueous levels of VEGF between Group A and Group B (*P* = 0.985).

**Table 1:** Main findings about the patient groups

	Mean Age (years)	Aqueous VEGF (pg/ml)	CRP (mg/ml)	HbA1c (%)	Fasting Glucose (mg/dl)
Group A Control (n: 18)	67.2 ± 13.5	62.6 ± 48.8 (8.4-228)	0.63 ± 0.59	5.5 ± 2.2	96.6 ± 7.8
Group B NDR (n: 14)	65.8 ± 10.8	56.5 ± 27.5 (28-114)	0.53 ± 0.45	13.1 ± 4.3	153.6 ± 48.8
Group C NPDR (n: 27)	63.7 ± 8.5	133.3 ± 58.5* (43-279)	0.79 ± 1.33	10.9 ± 2.5	195 ± 51.6
Group D PDR (n: 19)	65.1 ± 9.9	318.6 ± 238.6* (83-836)	0.59 ± 0.31	11.7 ± 3.6	188.2 ± 49.7

Values are expressed as mean ± standard deviation, Statistically significant differences are indicated with an asterisk, NDR: without diabetic retinopathy, NPDR: non-proliferated diabetic retinopathy, PDR: proliferative retinopathy, CRP: C-reactive protein, HbA1c: glycozilated hemoglobin

**Table 2:** Comparison of aqueous VEGF levels between patient groups (Bonferroni adjusted Mann-Whitney U test)

Groups compared	U	2 tailed P*
A (n = 18) vs. B (n = 14)	125.500	0.985
A (n = 18) vs. C (n = 27)	80.000	< 0.001*
A (n = 18) vs. D (n = 19)	13.000	< 0.001*
B (n = 14) vs. C (n = 27)	45.000	< 0.001*
B (n = 14) vs. D (n = 19)	9.000	< 0.001*
C (n = 27) vs. D (n = 19)	101.000	0.001*

*P* considered significant if less than 0.05/4 = 0.0125, Statistically significant differences are indicated with an asterisk

The mean central macular thickness as measured by OCT was 224.91 ± 26 μm (176 – 272) in Subgroup C1 (without CS-DME) and 387.5 ± 87.3 μm (293 – 561) in Subgroup C2 (with CS – DME); this difference was statistically significant (Mann-Whitney U test; two tailed *P* < 0.001).

**Figure 2:**
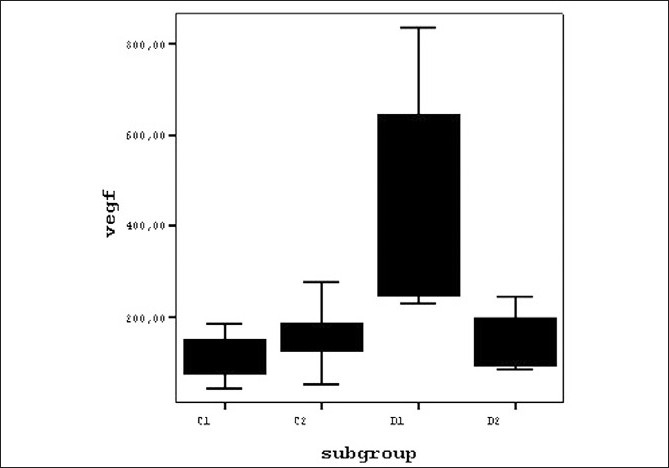
Diagram graphically displaying the differences between the aqueous VEGF levels of subgroups with diabetic retinopathy on boxplots C1= NPDR Subgroup without CSDME, C2= NPDR Subgroup with CSDME, D1= Active PDR Subgroup, D2= Quiescent PDR Subgroup

Statistical analysis of the aqueous VEGF levels of the NPDR subgroups showed a difference that was not significant statistically (Mann-Whitney U test; two-tailed *P* = 0,065), with comparable values in subgroup C1 (without CS-DME, 113.7 ± 49.63 pg/ml) and subgroup C2 (with CS-DME, 154.4 ± 61.32 pg/ml). On the other hand, a statistically significant difference (Mann-Whitney U test; two-tailed *P* < 0.001) was found between the aqueous VEGF levels of subgroups D1 (Active PDR, 466.18 ± 242.84 pg/ml) and D2 (Quiescent PDR, 143.12 ± 66.86 pg/ml) [Table 3 and Fig. 2].

**Table 3:** Data and comparison of subgroups with diabetic retinopathy

Subgroups	Sex (M/F)	Age (years)	VEGF (pg/ml)	Mann-Whitney U Test (Subgroup VEGF comparison)
C1(CSDME-) N = 14	5/8	64.1 ± 10	113.7 ± 49,6 (43.3 – 185.7)	U = 53.000 Two tailed *P* = 0.065
C2 (CSDME+) N = 13	4/8	64 ± 6.9	154.4 ± 61.32 (50.8 – 279)	
D1 (ACTIVE PDR) N = 10	6/4	68 ± 9.9	466.18 ± 242.84^*^ (229.6 – 836.1)	U = 4.000 Two tailed *P* = 0.001
D2(QUIESCENT PDR) N = 9	2/6	62.9 ± 9.8	143.12 ± 66.86^*^ (83 – 244.6)	

Values are expressed as mean ± standard deviation, Statistically significant differences are indicated with an asterisk, CSDME: clinically significant diabetic macular edema, PDR: proliferative retinopathy

We also analyzed the correlation between aqueous levels of VEGF and other variables of the three diabetic patient groups. The Pearson correlation test detected no statistically significant correlation between aqueous VEGF levels and serum levels of HbA1c (r = -0.003 *P* = 0.982), glucose (r = 0.214 *P* = 0.087) or CRP (r = -0.31 *P* = 0.784) for the three diabetic groups.

## Discussion

VEGF was originally named ‘Factor X’ by Michealson in 1948. More recent studies identified VEGF as the major cytokine in the pathogenesis of DR, showing a strong relationship between the increase in the intraocular VEGF levels and the development of PDR.[5–9] VEGF is also considered to play a key role in the pathogenesis of DME by causing increased vascular permeability.[10,11]

Data from several studies support the generally accepted supposition that the VEGF level in the aqueous liquid collected from the anterior chamber adequately reflects the VEGF activity in retinal tissues, despite a lower level of VEGF than in the vitreous liquid.[10] Based on these clinical data we investigated the relationship between the severity of DR and VEGF level by the way of aqueous collection. Previous clinical studies have shown that VEGF levels are elevated in the aqueous fluid and the vitreous gel of diabetic patients and that the severity of DR is strongly related to the VEGF levels detected in ocular fluids.[12–17] No correlation was found between the serum and aqueous VEGF levels in the previous studies, suggesting that the local VEGF production alone was responsible for the pathogenesis of DR.[14–16]

The main drawback of the present study was the small sample size of the subgroups making it difficult to interpret the results exhaustively. However, the findings of the present study show that:

1. The aqueous levels of VEGF elevated in eyes with DR compared to normal eyes and also to NDR eyes.
2. This elevation also correlated with the severity of DR, with a moderate increase of VEGF in the NPDR eyes and a more pronounced increase in active PDR eyes; eyes with quiescent PDR had lower VEGF levels, comparable to the levels in NPDR eyes.
3. NDR group eyes had VEGF levels similar to those of normal control eyes.
4. DME was associated with increased VEGF levels compared to eyes without DME, but the difference was not statistically significant.
5. There was a statistically significant difference between aqueous VEGF levels of the two PDR subgroups that helped to explain the therapeutic effectiveness of pan-retinal photocoagulation in PDR.

The elevation of VEGF levels was parallel to the severity of DR and to the degree of retinal ischemia suggesting that the main pathogenic factor causing VEGF elevation and responsible for DR progression in our patients’ eyes was retinal hypoxia.

Our results are in agreement with the results of the previous studies related to the major role of VEGF in the neovascular process in DR. Aqueous VEGF concentrations were moderately (two-fold) elevated in the group with NPDR, and the difference was statistically significant when compared to the control group and the group without DR. Although the increase in the aqueous VEGF concentration was very obvious (five-fold) in the group with PDR, an even greater increase (seven-fold) was observed in the active PDR subgroup compared to the control group and the group without DR.

There was statistically no significant difference between the aqueous VEGF levels of the control group and the diabetic group without DR. Considering also the lack of correlation between the VEGF and blood glucose, HbA1c, and CRP we presumed that the increase in ocular VEGF in DR was not associated with hyperglycemia, but mainly with hypoxia / ischemia in retinal tissues.

In our study, similar levels of aqueous VEGF that we observed in the two NPDR subgroups, eyes with and without CSME, are inconsistent with the results of the previous studies regarding the role of VEGF in DME and do not support the general opinion that DME is caused by a local increase in VEGF. A correlation between elevated VEGF in ocular fluids and the presence of DME has been revealed by some clinical studies, suggesting that VEGF is a mediator for hypoxic inflammation.[18–20]

However, the results of one study showed a limited clinical benefit of an intravitreal anti-VEGF agent (bevacizumab) in the treatment of diffuse DME, with no statistically significant increase in visual acuity and without normalization of the central macular thickness.[21] In addition, a further clinical study comparing the therapeutic effect of intravitreal triamcinolone and bevacizumab found that a corticosteroid (triamcinolone) was superior to the anti-VEGF agent (bevacizumab) in the treatment of DME, suggesting that the pathogenesis of DME could depend on factors besides VEGF.[22] Another clinical study showed a possible role of posterior hyaloidal traction in the development of DME, independent of VEGF.[18] Considering the complexity of DME pathogenesis and recognizing also the possible role of the posterior vitreoretinal interface in some DME cases, our results did not seem unreasonable. In spite of this, no posterior vitreoretinal traction was detectable on OCT in any eye from Group C of our study.

In conclusion, the present study shows that aqueous VEGF levels are correlated with the severity of DR and are significantly increased in patients with active PDR. These results emphasize that VEGF elevation is induced by retinal ischemia and plays a major role in the development and progression of PDR. However, an association between VEGF level and the development of DME is not apparent.
